# Does it take two to tango?

**DOI:** 10.1007/s12471-023-01845-5

**Published:** 2024-01-16

**Authors:** Daniel Mol, Erik A. Stel, Irene E. Hof

**Affiliations:** Department of Cardiology, OLVG Hospital, Amsterdam, The Netherlands

A 35-year-old athlete patient without any relevant medical history was referred to our electrophysiology laboratory because of exercise-induced palpitations. Due to the disabling complaints, the patient had to stop his marathon training. Cardiac echocardiography revealed a 47% ejection fraction and a left atrial volume index of 45 ml/m^2^. A previous exercise test showed an irregular heart rhythm which was suggestive of atrial ectopic beats and atrial fibrillation. We performed an electrophysiological study to induce and localise the atrial ectopic beats that may be the trigger for atrial fibrillation. The patient received general anaesthesia. During the isoprenaline washout phase, ‘irregularities’ became prominent, and the tracing displayed in Fig. 1 was recorded.

**Fig. 1:**
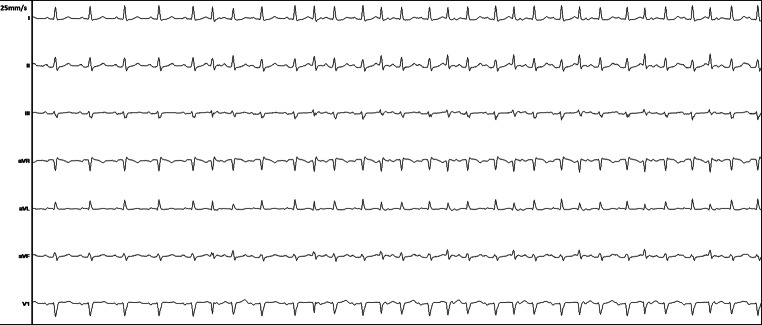
Seven-lead electrocardiogram recorded during the electrophysiological study

What would be your diagnosis for the rhythm displayed in Fig. 1?

## Answer

You will find the answer elsewhere in this issue.

